# Agora: An open science platform that enables evidence‐based exploration of Alzheimer's Disease therapeutic targets

**DOI:** 10.1002/alz70855_098626

**Published:** 2025-12-23

**Authors:** Jessica S Britton, Jesse C Wiley, Jaclyn Beck, Lawrence Yi, Beatriz Saldana, Brad Macdonald, Khai Do, Stockard Simon, Hallie Swan, Thomas Yu, Jay Hodgson, Anna Greenwood, Laura M Heath, Karina Leal

**Affiliations:** ^1^ Sage Bionetworks, Seattle, WA, USA

## Abstract

**Background:**

Agora (https://agora.adknowledgeportal.org) is an openly available web application developed to enable a broad spectrum of Alzheimer's disease (AD) researchers access to the target‐based evidence generated within the translational research portfolio of the National Institute on Aging (NIA). Agora accelerates AD research and maximizes therapeutic discovery by sharing information using interactive tools, data visualizations, and summarized evidence.

**Methods:**

Agora enables sharing information about potential AD therapeutic targets by surfacing data visualizations, aggregating summary evidence, and displaying results from targeted validation studies. Agora users can browse a list of over 950 potential therapeutic targets that have been nominated by the NIA's Accelerating Medicines Partnership in AD (AMP‐AD) consortium and Target Enablement to Accelerate Therapy Development for AD (TREAT‐AD) centers, and by the broader AD research community; see visualizations and summarized information based on harmonized genome‐wide analyses of high‐dimensional human transcriptomic, proteomic, and metabolomic data; use interactive tools designed to enable non‐bioinformaticians to evaluate and compare complex multi‐omic data; leverage results that objectively rank and organize targets based on their role in AD; investigate the experimental reagents and bioinformatics packages that are available to support further investigation of select targets; and access the underlying data that drives the results and visualizations presented in the application.

**Results:**

Agora presents information and results about AD targets using approaches that make this information accessible to a broad spectrum of AD researchers. Recent updates to Agora include 1) new TREAT‐AD target nominations and 2) enhanced connections to external resources, including the introduction of a new drug development resource section and UniprotKB integration.

**Conclusions:**

Advancing potential AD therapeutics through the drug development pipeline requires efforts from research groups and specialties spanning both academia and industry. Agora enables the AD research community to unite by enabling the development and sharing of target hypotheses to accelerate the investigation of promising new AD therapeutic targets, pathways and mechanisms.

